# Production of Fe nanoparticles from γ-Fe_2_O_3_ by high-pressure hydrogen reduction

**DOI:** 10.1039/d0na00635a

**Published:** 2020-08-26

**Authors:** I. Dirba, C. A. Schwöbel, A. Zintler, P. Komissinskiy, L. Molina-Luna, O. Gutfleisch

**Affiliations:** Functional Materials, Institute of Materials Science, Technical University of Darmstadt Alarich-Weiss-Straße 16 64287 Darmstadt Germany imants.dirba@tu-darmstadt.de; Advanced Electron Microscopy Division, Institute of Materials Science, Technical University of Darmstadt Alarich-Weiss-Straße 2 64287 Darmstadt Germany; Advanced Thin Film Technology, Institute of Materials Science, Technical University of Darmstadt Alarich-Weiss-Straße 2 64287 Darmstadt Germany

## Abstract

In this work, the reduction of iron oxide γ-Fe_2_O_3_ nanoparticles by hydrogen at high pressures is studied. Increasing the hydrogen pressure enables reduction of γ-Fe_2_O_3_ to α-Fe at significantly lower temperatures. At low pressures, a temperature of 390 °C is necessary whereas at 530 bar complete reduction can be realized at temperatures as low as 210 °C. This leads to significant improvement in the final particle morphology, maintaining high surface-to-volume ratio of the nanoparticles with an average size of 47 ± 5 nm which is close to that of the precursor γ-Fe_2_O_3_. Neck formation, coalescence and growth during reduction can be significantly suppressed. Investigations of magnetic properties show that saturation magnetization of the reduced α-Fe nanoparticles decreases with particle size from 209 A m^2^ kg^−1^ at 390 °C reduction temperature to 204 A m^2^ kg^−1^ at 210 °C. Coercivity for the fine iron particles reaches 0.076 T which exceeds the theoretical anisotropy field. This is attributed to nano-scale surface effects.

## Abstract 2

In this work, the reduction of iron oxide γ-Fe_2_O_3_ nanoparticles by hydrogen at high pressures is studied.

## Graphical abstract

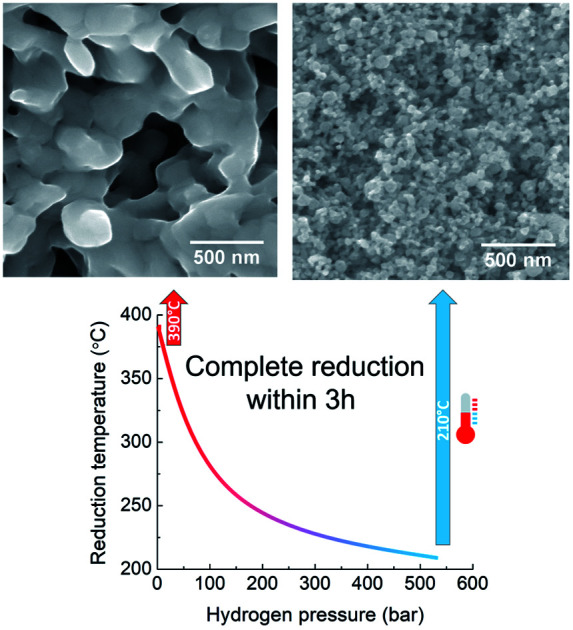

## 1. Introduction

Iron nanoparticles have important applications due to their unique chemical and magnetic properties.^1^ For example, it has been shown that compared to micrometer-sized particles, the catalytic activity of nanostructured iron for the Fischer–Tropsch process is much higher due to the larger surface area.^2^ Similar catalytic activity to platinum-based materials for chemical hydrogen storage applications has been reported for amorphous Fe nanoparticles.^3^ It has also been demonstrated by Dirba *et al.* that fine Fe nanoparticles are crucial for formation of the α′′-Fe_16_N_2_ phase using low-temperature nitriding with ammonia.^4^ Furthermore, since iron has the highest saturation magnetization among all ferromagnetic elements, yet is a cheap, abundant and environmentally friendly material, numerous applications of Fe nanoparticles in magnetism are promising. Exchange-coupled nanocomposite magnets for remanence and maximum energy product enhancement, consisting of nanoscale α-Fe as the magnetically soft phase and SmCo_5_, Nd_2_Fe_14_B or Sm_2_Fe_17_N_3_ as the hard phases, have been investigated.^5–8^ Furthermore, the potential in biomedical applications, such as magnetic resonance imaging^9^ or hyperthermia mediated drug release for cancer therapy^10^ has also been discussed if a proper surface coating (*e.g*. Au) is provided.^11^

Synthesis of nanoparticles with controlled particle size, morphology and surface modifications can be achieved by solution chemistry techniques.^12–14^ This approach is well suited for biomedical applications where minimizing reactivity and agglomeration is important. However, for magnetic nanocomposites and catalysts, the presence of an organic shell around the nanoparticles is not favorable, because it competes with the exchange length (<5 nm for typical hard magnetic materials; *e.g*. 1.9 nm in for Nd_2_Fe_14_B^15^) in the former and hinders active surface sites for the latter. Therefore, in this work we demonstrate how to produce fine α-Fe nanoparticles using a gas–solid high-pressure hydrogen reduction process.

In literature, reduction of iron oxides to α-Fe with hydrogen is typically conducted at temperatures of at least 390 °C–500 °C.^16–18^ This choice of temperature is supported by hygrometry measurements, where the maximum rate of H_2_O production was from 389 °C to 522 °C^19,20^ depending on the heating rate. Heat treatment at such elevated temperatures leads to particle coalescence and growth of their effective size, followed by a detrimental reduction of their surface area due to temperature-enhanced diffusion which increases exponentially with temperature. A sintering prevention layer, such as Al_2_O_3_,^21^ hydroxyapatite^22^ or carbon^23^ can be helpful, which, however, covers the produced nanoparticles and, therefore is also not suitable for catalyst or exchange-coupled nanocomposite applications due to the additional shell.

Assuming a single-step reaction, hydrogen reduction of Fe_2_O_3_ can be written as1
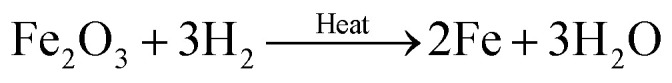
With a reaction constant *K*_r_ = *p*_H_2_O_^3^/*p*_H_2__^3^, where *p*_H_2_O_ and *p*_H_2__ are the partial pressures of water vapor and hydrogen respectively. By using the thermodynamic data from ref. 24, the calculated reaction Gibbs free energy of Δ*G*^0^ = 55 kJ mol^−1^ and enthalpy of Δ*H*^0^ = 98 kJ mol^−1^ are obtained. Thus, the reaction (1) is endergonic and endothermic, and does not occur spontaneously at ambient conditions. Taking the expression for *K*_r_ into account, a shift of the chemical equilibria to the right (towards products) and complete reduction of the iron oxide to Fe at lower temperatures is achievable at higher hydrogen partial pressure *p*_H_2__. Therefore, in this work, we attempt to achieve complete reduction of Fe_2_O_3_ at significantly lower temperatures by using high hydrogen pressures. This approach should result in improved morphology of the produced nanoparticles since the detrimental coarsening during the reduction step is suppressed.

## 2. Experimental

Commercial maghemite γ-Fe_2_O_3_ nanoparticles from Alfa Aesar with an average particle size of 20–40 nm according to the manufacturer's datasheet, were used as a precursor. Reduction experiments were done in a hydrogen (99.999% purity, Linde) atmosphere in a custom-made autoclave system equipped with high pressure/high temperature vessel (series 4740, Parr Instrument Company) and an external vertical tube heater assembly (Model 4921, Parr Instrument Company). The working volume of the vessel was 75 cm^3^. 0.050 g of iron oxide nanoparticles were filled in a steel crucible and mounted in the pressure vessel. After twice evacuating and purging with Ar (99.999% purity, Air Liquide), the reactor was heated to target temperature under continuous pumping and then pressurized with hydrogen. The reduced samples were handled in an Ar filled glovebox (*p*(O_2_) < 0.1 ppm, MBraun) in order to avoid oxidation.

Magnetic properties of the nanoparticles were measured using vibrating sample magnetometer (VSM) (7400 Series, Lake Shore). Microstructural investigations were carried out using a scanning electron microscope (SEM, Philips XL30 FEG, 15 kV acceleration voltage). For scanning transmission electron microscopy (STEM) a JEOL JEM 2100F was used. The microscope was operated at 200 kV. The sample was prepared on a lacey carbon Cu grid in a glovebox and transferred to the TEM in a Gatan 648 double tilt vacuum transfer holder under nitrogen atmosphere to prevent oxidation. In addition to high-resolution (HR) TEM imaging and energy dispersive X-ray spectroscopy (EDS) in scanning transmission electron microscopy (STEM), nanobeam diffraction was performed to acquire electron diffraction data from different regions of the particle. The full width half maximum of the electron beam was 2 nm. Crystal structure and phase purity of the nanoparticles were investigated by X-ray diffraction (XRD) using a powder diffractometer (STOE, Stadi P) with Mo K_α1_-radiation (*λ* = 0.70930 Å) in Debye–Scherrer geometry with powders sealed in quartz capillaries to avoid oxidation. X-ray photoelectron spectroscopy (XPS) measurements were performed on compacted nanoparticle pellets using a PHI Versaprobe 5000 spectrometer with monochromatic Al K_α_ radiation.

## 3. Results and discussion

**Fig. 1:**
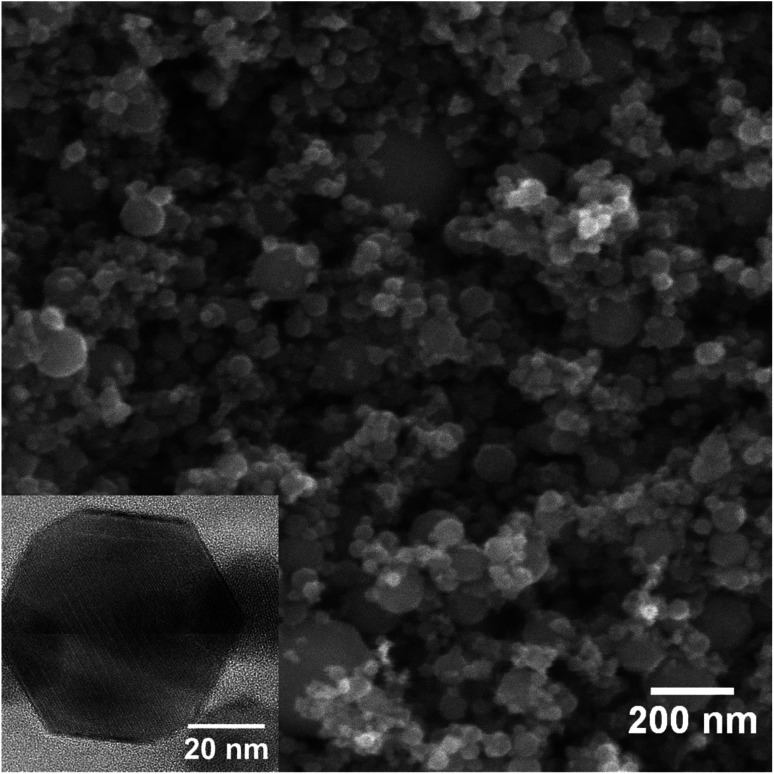
SEM image of the initial γ-Fe_2_O_3_ nanoparticles. The inset shows TEM image of a single nanoparticle.

The initial γ-Fe_2_O_3_ is a typical brown rust-like powder. The average particle size is expected to be around 20–40 nm according to manufacturer's data. SEM and TEM images of the powder are presented in Fig. 1. Particles show a broad size distribution from fine 20 nm nanoparticles to several large particles in the range of hundreds of nanometers. The average particle size estimated from the SEM images is 52 ± 31 nm (152 particles counted). The average crystallite size obtained from XRD peak broadening is 55 ± 5 nm. The TEM image shows that the fine nanoparticles mostly have well-defined hexagonal shapes.

**Fig. 2:**
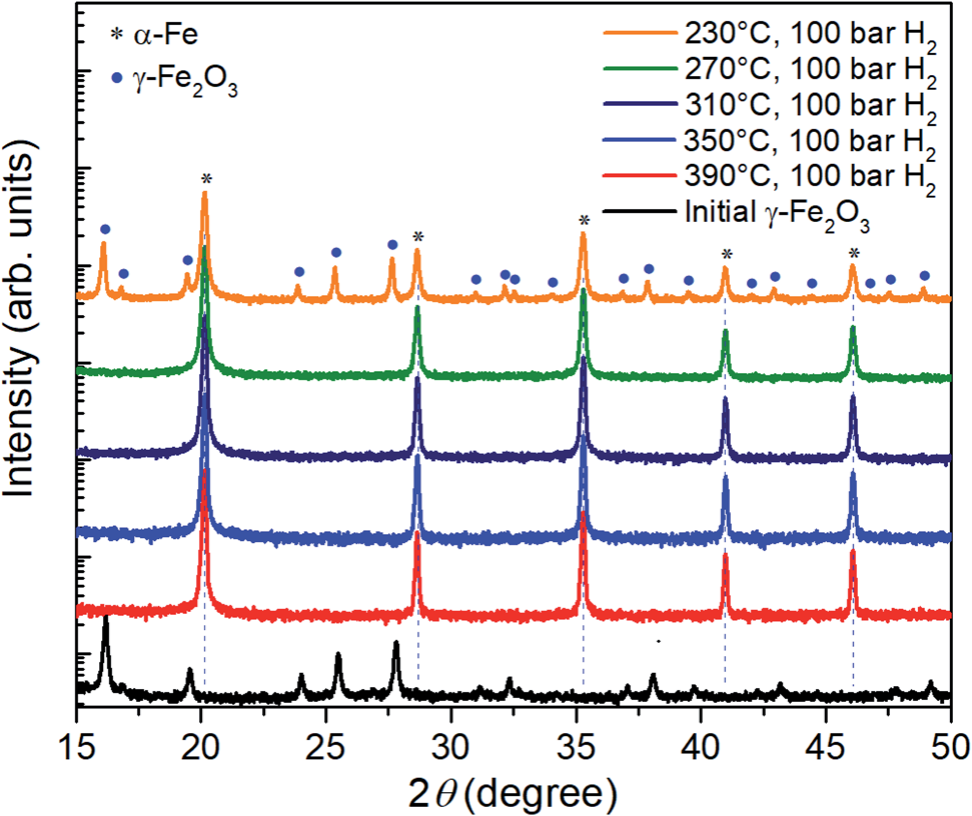
XRD patterns of the γ-Fe_2_O_3_ nanoparticle powder before and after three-hour reduction in hydrogen at a pressure of 100 bar and temperatures between 230 °C and 390 °C.

Fig. 2 shows XRD patterns of the initial γ-Fe_2_O_3_ and the same nanoparticles reduced at a hydrogen pressure of 100 bar for 3 hours at different temperatures in the range from 230 °C to 390 °C. At 390 °C, in agreement to previous reports, reduction can be achieved even at low pressures (not shown here). 100 bar hydrogen pressure is sufficient for complete reduction at temperatures down to 270 °C within 3 h. Further lowering of the reduction temperature to 230 °C leads to partial reduction and remaining γ-Fe_2_O_3_ reflections are evident in the XRD data.

**Fig. 3:**
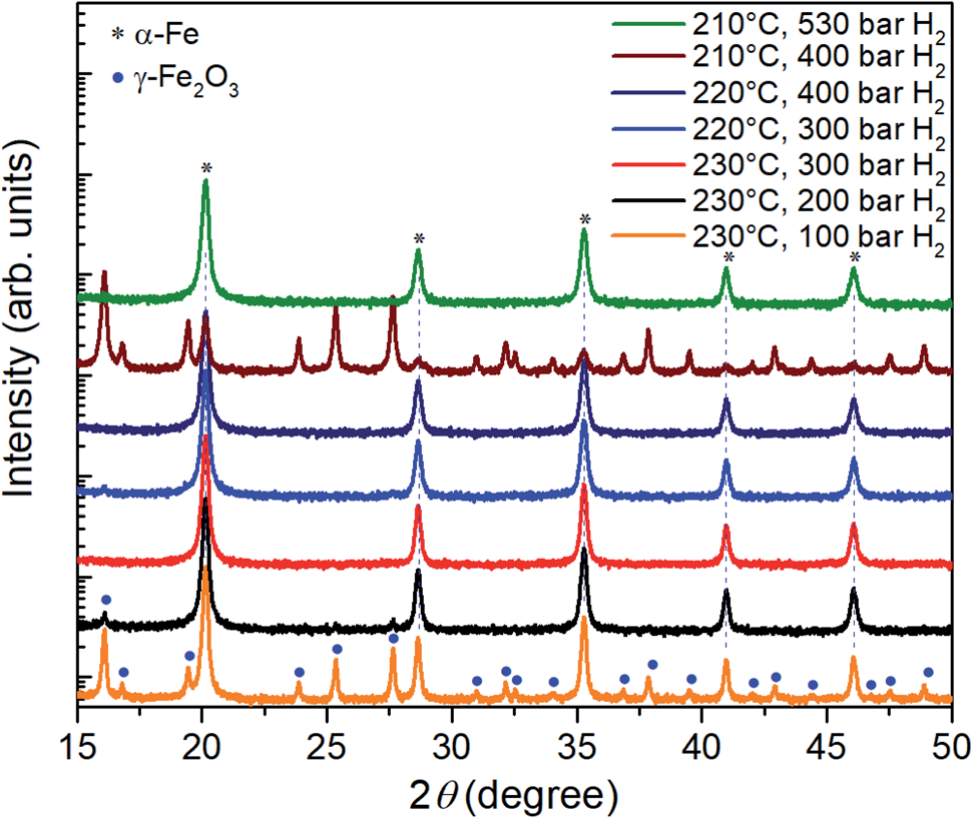
XRD patterns of the γ-Fe_2_O_3_ nanoparticle powders after three-hour reduction at temperatures of 210°C–230 °C and hydrogen pressures in the range between 100 bar and 530 bar.

In a next step, the reduction of γ-Fe_2_O_3_ nanoparticles at temperatures below 230 °C was investigated at hydrogen pressure above 100 bar. The corresponding XRD results are presented in Fig. 3. At 230 °C and 200 bar hydrogen pressure, a small signal from the highest intensity iron oxide diffraction peak can be identified at 16.1°.

Therefore, the pressure was increased further to 300 bar at which the γ-Fe_2_O_3_ nanoparticles are fully reduced; only reflections from α-Fe are visible in the corresponding diffractogram. At lower temperatures of 220 °C and 210 °C, a hydrogen pressure of 400 bar and 530 bar is necessary to complete the reduction in 3 hours. Thus, by increasing the hydrogen pressure to 530 bar, it is indeed possible to reduce the temperature necessary for complete reduction of γ-Fe_2_O_3_ nanoparticles to α-Fe from 390 °C to 210 °C. The pressure of 530 bar was the limit for the autoclave, a further decrease in the reduction temperature for higher pressures can be expected. On the other hand, it is possible that at a certain pressure the benefits from the lower temperature will be outweigh by a reduction of the diffusion barrier^25^ and gas-pressure sintering effects.^26^

**Fig. 4:**
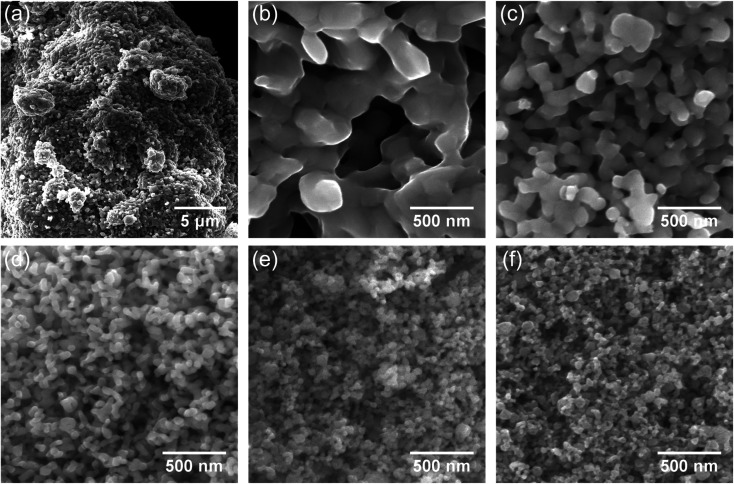
SEM images of the hydrogen reduced nanoparticles. (a) Low and (b) high magnification images of a sample reduced at 390 °C (100 bar). (c–f) High magnification SEM images of samples reduced at 310 °C (100 bar), 270 °C (100 bar), 230 °C (300 bar) and 210 °C (530 bar) respectively.

In order to investigate the coalescence and growth of the nanoparticles at the proposed high-pressure, low-temperature hydrogen reduction, free-standing nanoparticles were examined using SEM. The reduction at 390 °C leads to severe bonding of the nanoparticles into sponge-like agglomerates with sizes in several tens of micrometers (Fig. 4a). A coalescence of the nanoparticles into large grains with much smaller surface area is observed in the high-resolution SEM image in Fig. 4b. Bridging between neighboring nanoparticles and their coalescence occur also at 310 °C (Fig. 4c) and at 270 °C (Fig. 4d), although, with much smaller increase in the size. However, after reduction at 230 °C (Fig. 4e) and especially at 210 °C (Fig. 4f), neck formation and coalescence are minimized, and individual nanoparticles can be distinguished. Thus, the coalescence of the nanoparticles during the reduction process has been suppressed. Clustering still takes place due to magnetostatic interactions, therefore monodisperse particles could not be achieved. The average size of the obtained phase-pure α-Fe nanoparticles is comparable with that of the γ-Fe_2_O_3_ precursor. A particle size analysis was conducted by the line interception method in which an average size was calculated for the grains along 4 lines (horizontal, vertical and both diagonals)^27^ in the respective SEM images. The obtained sizes of the nanoparticles are summarized in Table 1, including a comparison with the crystallite size, extracted from the XRD peak broadening. Usage of lower reduction temperatures is clearly beneficial and results in fine α-Fe nanoparticles with a size smaller than 50 nm. Clearly, lowering of the reduction step temperature results in much finer α-Fe particles. Error in the SEM data is caused mainly by a limited number and inaccurate size measurements of the analyzed nanoparticles in the SEM images, especially for large concrescent particles at high reduction temperatures.

**Table 1:** Particle and crystallite sizes extracted from the SEM and XRD measurements, respectively, of the α-Fe nanoparticles obtained by reduction at different temperatures and hydrogen pressures

Temperature (°C)	Particle size (nm)	Crystallite size (nm)	Hydrogen pressure (bar)
390	215 ± 37	263 ± 46	100
310	96 ± 47	133 ± 12	100
270	67 ± 2	79 ± 1	100
210	47 ± 5	41 ± 1	530

**Fig. 5:**
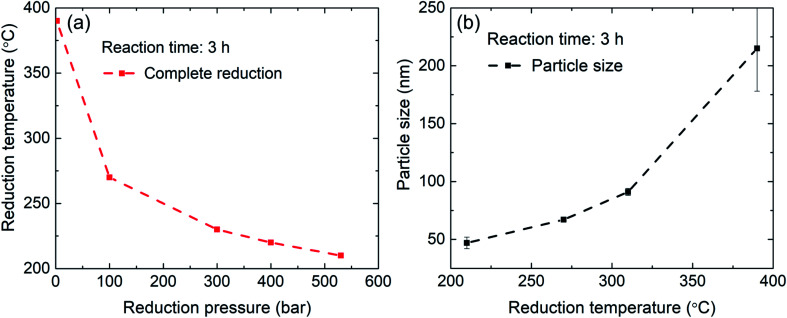
Summary of the results from hydrogen reduction experiments. (a) Gradual increase in hydrogen pressure enables lowering of the reaction temperature. (b) At lower reduction temperatures particle growth is minimized.

The extracted value of the crystallite size is additionally influenced by the broadening of the XRD reflections due to microstrain. Fig. 5 summarizes the results of hydrogen reduction experiments. The data points represent temperature–pressure pairs where complete reduction of Fe_2_O_3_ to α-Fe was achieved according to the XRD data. It can be concluded that reduction of iron oxide nanoparticles at elevated hydrogen pressures and consequently lower temperatures can indeed be used for production of high-quality iron nanoparticles without the detrimental particle coarsening.

**Fig. 6:**
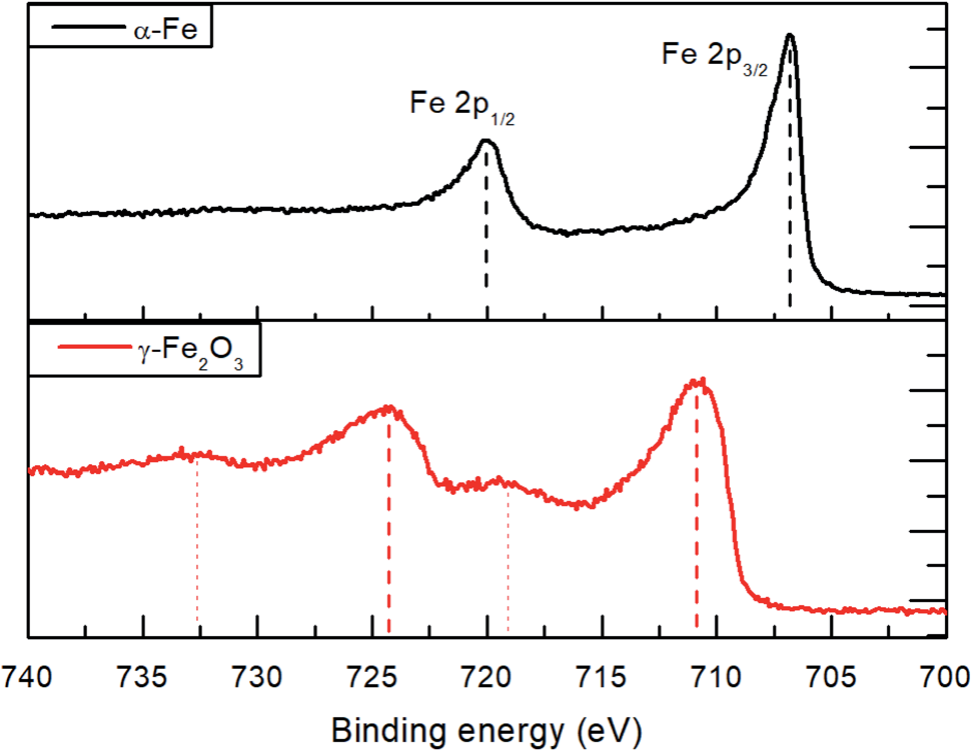
XPS Fe2p spectra of the initial γ-Fe_2_O_3_ powder and α-Fe nanoparticles produced by reduction in hydrogen atmosphere at 530 bar and 210 °C.

Due to the broadening of the XRD reflections in nanoparticles and a possibility that some fraction of the material is amorphous, small amounts of residual iron oxide could remain unnoticed in the XRD data. For this reason, XPS measurements of the initial γ-Fe_2_O_3_ powder and the reduced α-Fe nanoparticles were conducted to analyze electronic structure of Fe (Fig. 6). Prior to the XPS measurements, the samples were sputtered for 10 minutes using Ar^+^ ions at 2 kV to remove the adventitious carbon contamination and oxidized layers on the sample surface due to contacts with atmospheric oxygen.

The Fe2p spectra for the initial γ-Fe_2_O_3_ nanoparticles show emissions at 710.8 and 724.5 eV (a spin–orbit splitting of 13.7 eV) for the Fe2p_1/2_ and Fe2p_3/2_, respectively. The observed shake-up satellite peaks are shifted from the parent peaks by 8.5 eV. Within the measurement accuracy, the spectra correspond to those reported in literature for γ-Fe_2_O_3_.^28,29^ The Fe2p spectra of the obtained α-Fe nanoparticles reveal a textbook-like^30^ Fe2p_1/2_ and Fe2p_3/2_ emissions at 706.8 and 719.9 eV, respectively, with 13.1 eV spin–orbit splitting and absence of shake-up satellites. The XPS results indicate complete reduction of the γ-Fe_2_O_3_ powder at 210 °C and 530 bar hydrogen pressure. It has to be noted that the measured initial survey spectra show oxygen and carbon on the surface which can be removed after *in situ* Ar ion sputtering. This indicates surface oxidation of the α-Fe nanoparticles.

**Fig. 7:**
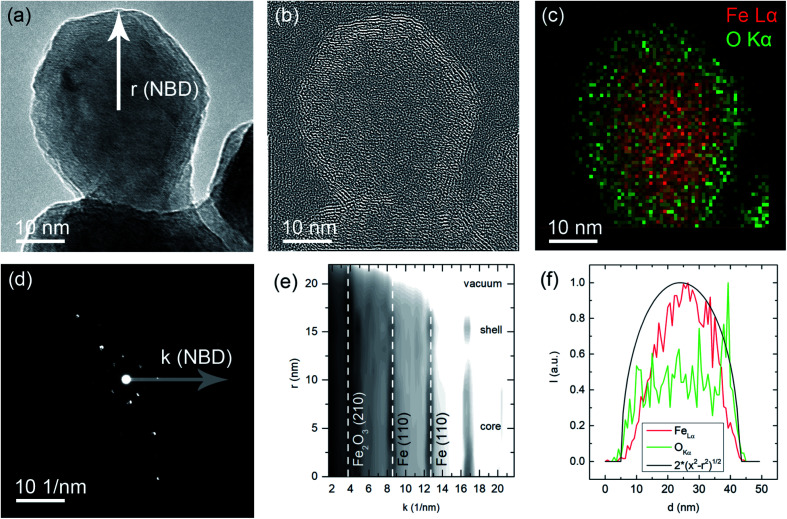
(a) HRTEM image of a representative nanoparticle, the NBD scan direction is indicated originating at the center of the particle. (b) Bandpass/Fourier filtered image with the Fe_2_O_3_ (210) distance selected, note that this lattice spacing is only observed for the shell of the particle. (c) Color-coded EDS map of the particle (red: Fe–La, green O–K). (d) Exemplary NBD electron diffraction pattern with the radius in *k*-space indicated. (e) Intensity profile of rotational averaged NBD patterns recorded along the scan line indicated in (a). Note that for the center of the particle, the intensities of the Fe lattice spacings are prominent and for the edge/shell of the particle the Fe_2_O_3_ lattice spacings are stronger. (f) Line profiles of the characteristic line intensities plotted in (c). An ideal distribution for a homogeneous and spherical particle is also plotted.

In the HRTEM image shown in Fig. 7a a spherical particle shape can be observed in accordance to the SEM imaging discussed above. A core–shell type oxidation layer is revealed. Since atomic resolution information is included in this image, it is possible to apply a bandpass filter for the lattice spacing of the Fe_2_O_3_ (210) lattice spacing (*d* = 2.519 Å). The filtered image shown in Fig. 7b clearly shows the occurrence of this spacing in the outer or shell regions of the particle. This coincides with increased oxygen concentration in the shell, which is evident in the STEM-EDS mapping, Fig. 7c. As a spatially resolved electron diffraction (ED) method, NBD allows for the collection of *k*-space data with a high real space resolution. A series of NBDED patterns was acquired along indicated line in Fig. 7a and the azimuthal integrated intensities are plotted in Fig. 7e. Characteristic Bragg intensities are marked for both phases, α-Fe and γ-Fe_2_O_3_, it can be observed that the core of the particle, the Fe intensities are prominent and with increasing distance from the center of the particle, the Fe_2_O_3_ phase intensities increase. In the experimental geometry, only a projection can be observed, which is causing a convolution of the probed volume in both the NBD patterns and the EDS intensities. This behavior is shown in Fig. 7f, where a homogeneous distribution of an element in a spherical particle is indicated. As discussed above, the core–shell structure of the particle becomes evident with the increased concentration of Fe in the center of the particle and with the increased O content for the shell, both deviating from the ideal distribution. These results are in agreement with the XPS survey measurements and show that surface oxidation during handling could not be fully avoided.

**Fig. 8:**
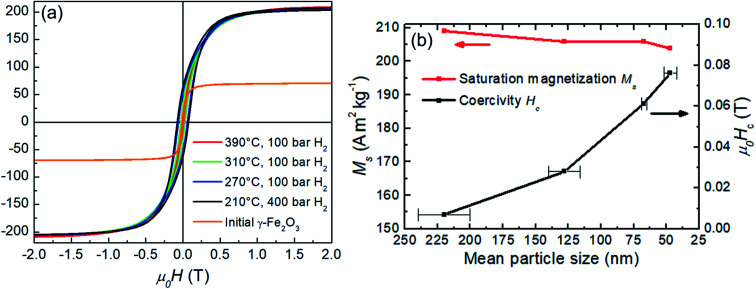
(a) Hysteresis loops for the initial γ-Fe_2_O_3_ and α-Fe particles produced by hydrogen reduction at 390 °C, 310 °C, 270 °C and 210 °C. (b) Saturation magnetization *M*_s_ and coercivity *H*_c_ at room temperature as a function of the particle size for the hydrogen reduced α-Fe nanoparticles.

Magnetic properties of the precursor Fe_2_O_3_ and hydrogen reduced Fe nanoparticles are summarized in Fig. 8. Room temperature *M*(*H*) loops for pure samples reduced at 390 °C, 310 °C, 270 °C and 210 °C are shown in Fig. 8a with the corresponding *M*_s_ and *H*_c_ values as a function of the size of the reduced particles, plotted in Fig. 8b. The initial γ-Fe_2_O_3_ powder shows *M*_s_ = 70 A m^2^ kg^−1^ which is slightly lower than the bulk value of 74 A m^2^ kg^−1^.^31^ There is a decrease in *M*_s_ with particle size for the α-Fe samples from 209 A m^2^ kg^−1^ (390 °C reduction temperature) to 204 A m^2^ kg^−1^ (210 °C reduction temperature). Similar findings have been reported by Hsu *et al.*,^32^ where the lower saturation magnetization was explained by the semi-infinite nature of fine particles. The atoms on the free surface of nanoparticles possess a lower magnetization than the bulk material due to the lack of translational symmetry and broken magnetic exchange bonds on the surface. For this reason, finer particles lead to reduced *M*_s_ due to the large surface area. Another contributing factor to the reduced magnetization as a function of the reciprocal particle size is also the slight surface oxidation during handling and measurement as evident from the TEM and XPS data.

Coercivity *H*_c_ scales inversely with the particle size and increases more than 10-fold from 0.0069 T (390 °C reduction temperature) to 0.076 T (390 °C reduction temperature). The increased coercivity for smaller particles (lower reduction temperatures) is consistent with the expectation that the nucleation field is maximum for single-domain particles.^33^ For a spherical iron particle, the single-domain diameter is about 10 nm,^15^ which is smaller than the experimental particle size. Thus, a further coercivity enhancement for even finer particles can be expected and indeed *H*_c_ values of 0.106 T for the mean particle size of approximately 30 nm have been reported.^32^ According to the Stoner–Wohlfarth model,^34^ the maximum coercivity is defined by the anisotropy field *H*_a_ = 2*K*_1_/*μ*_0_*M*_s_, where *K*_1_ is the principal anisotropy constant. For iron, *K*_1_ ≈ 50 kJ m^−3^, *μ*_0_*M*_s_ ≈ 2.15 T and, therefore *H*_a_ ≈ 0.06 T. However, typically the measured coercivities reach only small fraction, approximately 25% of the theoretical *H*_a_ which is known as the Brown's paradox.^35,36^ Interestingly, as demonstrated in the discussion above, coercivity of fine Fe particles exceeds the calculated theoretical upper limit – the anisotropy field *H*_a_. Therefore, most likely additional surface-related phenomena take place and account for the discrepancy. For example, an increase in the anisotropy constant with the reciprocal particle diameter up to 300 kJm^−3^ for 2 nm α-Fe nanoparticles has been reported^37^ and is attributed presumably to influence of surface effects. Also, the observed surface oxidation may play a role due to exchange coupling between the core/shell layers.

## 4. Summary

In this work, we have demonstrated that by increasing the hydrogen pressure up to 530 bar, it is possible to lower the temperature necessary for complete reduction of γ-Fe_2_O_3_ nanoparticles to α-Fe from 390 °C down to 210 °C. This significant improvement in reduction temperature was shown to be beneficial for the final particle morphology. Coalescence and sintering of the particles accompanied by surface area loss which occurs at elevated temperatures (*e.g*. 390 °C), can be suppressed when reduction is performed at 210 °C. The saturation magnetization of the reduced α-Fe nanoparticles decreases with particle size from 209 A m^2^ kg^−1^ at 390 °C reduction temperature to 204 A m^2^ kg^−1^ at 210 °C. Coercivity shows opposite behavior and even exceeds the theoretical anisotropy field for the fine 47 nm particles. This is attributed to surface effects. TEM investigations reveal that the Fe nanoparticles are passivated with a Fe_2_O_3_ layer resulting in a core–shell structure.

These findings are also relevant for applications such as catalysis and exchange-coupled nanocomposites, where fine iron nanoparticles with high surface area are required. The presented method can be extended to other metal-oxide systems.

## Conflicts of interest

There are no conflicts to declare.

## Supplementary Material
